# 665. Duration of Spontaneous Bacterial Peritonitis Prophylaxis in Acute Variceal Hemorrhage Patients

**DOI:** 10.1093/ofid/ofad500.728

**Published:** 2023-11-27

**Authors:** Carissa M Tedeschi, Abigail Hoff, Nicole Scherrer, Amanda Shigle, Sheena Burwell

**Affiliations:** WVU Medicine – WVU Hospitals, Morgantown, West Virginia; UCHealth, Cincinatti, Ohio; WVU Medicine – WVU Hospitals, Morgantown, West Virginia; WVU Medicine – WVU Hospitals, Morgantown, West Virginia; West Virginia University Medicine, Morgantown, West Virginia

## Abstract

**Background:**

To prevent spontaneous bacterial peritonitis (SBP), re-bleeding, mortality, and long hospital stays in patients with acute variceal hemorrhage, the American Association for the Study of Liver Diseases recommends up to 7 days of ceftriaxone, with guidance to consider discontinuing when hemorrhage resolves and vasoactive drugs are stopped. Due to the ambiguous recommendation, practices vary surrounding duration of SBP prophylaxis. To our knowledge, no study has been completed on the duration of SBP prophylaxis within our current standard of care.

**Methods:**

This was a retrospective, non-inferiority trial of adult patients receiving an octreotide infusion with confirmed variceal hemorrhage diagnosed via esophagogastroduodenoscopy at WVU Hospitals from 1/1/2020 - 12/31/2022. Medical records were reviewed to include demographic information, antibiotic selection, and duration of therapy for SBP prophylaxis, in addition to outcome measures such as infection, re-bleeding, length of stay, and mortality. Patients who received 5 or fewer days (shorter group) of prophylactic antibiotics were compared to those who received more than 5 days (longer group).

**Results:**

84 patients were included: 39 patients in the shorter group, and 45 patients in the longer group. There was no statistically significant difference found between the shorter and longer groups in terms of demographic information. The primary outcome was development of infection. Four (10.3%) patients in the shorter group developed infection, and three (6.7%) patients in the longer group developed infection. The number of patients gathered met power for a 20% non-inferiority margin. There was no statistically significant difference found in any of the secondary outcomes.

Results

**Figure:**
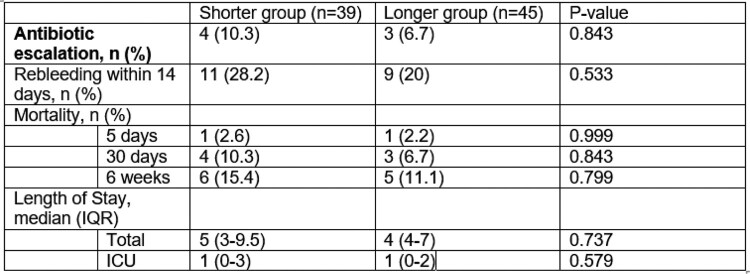

Primary and Secondary Outcomes

**Conclusion:**

We can conclude that shorter durations of SBP prophylaxis are non-inferior to longer durations for preventing infections. In general, decreasing the duration of antibiotics to only what is necessary allows for preventing the development of resistance and fewer secondary infections. Additional studies are needed to confirm the results and impact guideline recommendations.

**Disclosures:**

**All Authors**: No reported disclosures

